# Effect of khat extract on color stability of digitally and manually fabricated provisional restorations: an in vitro comparative study

**DOI:** 10.1186/s12903-023-03425-w

**Published:** 2023-09-29

**Authors:** Abd Al-Rhaman M. Al-Akhali, Mohsen Al-Hamzi, Ibrahim Z. Al-Shami, Abdulwahab I. Al-Kholani, Ahmed A. Madfa

**Affiliations:** 1 Department of Conservative Dentistry and Fixed Prosthodontics, Faculty of Dentistry, Sana’a University, Sana’a, Yemen; 2 Department of Restorative Dental Science, College of Dentistry, University of Ha’il, Ha’il, Kingdom of Saudi Arabia; 3 Department of Conservative Dentistry, Faculty of Dentistry, Thamar University, Thamar, Yemen

**Keywords:** Poly-methyl methacrylate (PMMA), Provisional Restoration (PR), CAD/CAM PR, 3D Printing PR, Khat extract, Color stability

## Abstract

**Background:**

The aim of this in vitro study was to evaluate the effect of khat extract on the color stability of five different provisional restorative materials (PRMs).

**Methods:**

In this study, 50 specimens were fabricated from five different PRMs with different techniques. Twenty specimens were digitally fabricated of poly-methyl methacrylate (PMMA) CAD/CAM milling and 3D printing PRMs, while the other thirty specimens were manually fabricated of three different PRMs: PMMA self-cured (SC) acrylic resin, light-cured (LC) composite, and Bis-acrylic SC composite. Milling and 3D printing machines were used to fabricate the digital specimens, while the manual specimens were fabricated using a metallic mold. The material was placed in the mold, covered by a polyester stripe, and held between two glass slabs with a constant load for 30 s. After setting, the specimens were removed and checked. Ten disc-shaped specimens with 2 ± 0.3 mm thickness and 10 ± 0.3 mm diameter were prepared from each of the tested PRM. Then all the specimens were polished. Five specimens of each PRM were immersed in khat extract, while the other five were immersed in distilled water medium as a control group. The color measurements were recorded before and after 1 and 7 days of immersion using a spectrophotometer. The immersion media were renewed every 24 h and kept along with the specimens at 37 °C. The T test, paired T test, and ANOVA analysis of variance were used to analyze the results. The Bonferroni test was used for post-hoc multiple comparisons.

**Results:**

The interaction between the tested PRMs, the media, and the duration of immersion time was statistically significant (p < 0.05). PMMA CAD/CAM milling PRM was the most stable in color, and this was statistically significant (p < 0.05). The LC composite PRM composite was the least stable in color and was statistically insignificant (p > 0.05) when compared to the 3D printing and Bis-acrylic SC composite PRMs, respectively.

**Conclusions:**

This study demonstrated that khat extract medium has a high staining ability on the tested PRMs. CAD/CAM milling PRM was the most stable in color and could therefore be used as a long-term provisional. The increase in immersion time was a significant factor in the color change of the tested PRMs. The color of the 3D-printed PRM was the most affected over time.

## Introduction

A provisional restoration (PR) now serves more as a diagnostic tool than just a space maintainer. In dentistry, provisional or interim restorations are often used between the preparation of the tooth and the placement of the definitive prosthesis [1]. While the definitive prosthesis is being made in the dental laboratory, which typically takes around 7 to 10 days, PR allows the patient to perform various biological, masticatory, esthetic, and speech functions [2]. Although each of these goals is crucial, the patient frequently places the most emphasis on the PR’s aesthetics, particularly when the PR will be worn for an extended amount of time or is in the aesthetic zone [3–7]. Furthermore, in clinical settings such as changes in the vertical dimension in full oral rehabilitation, immediate load implant prosthetics, long-span fixed prostheses, therapies for temporomandibular joint dysfunction, or patients who exhibit para-functional habits, the mechanical properties of PRs play an important role in enabling the dentist to critically evaluate commercial products and select the best material for a given clinical situation during the provisional prosthesis phase [8, 9].

By properly managing this intermediate stage of therapy, dentists can gain the confidence of their patients [8]. However, discoloration of provisional restorations (PRs) may make patients unhappy [4, 10]. Even though color stability is just one factor to consider when selecting a provisional material, patients and dentists who work in the esthetic zone place a high value on it [11].

Different materials are currently available for fabricating PRs. Most of these materials are made of methacrylate resin or Bis-acrylate composite resin [12]. Poly-methyl Methacrylate (PMMA), a heat-processed thermosetting material, was first introduced in 1936 [13]. It was quickly adapted for use in dentistry as a self-curing prosthetic and restorative resin [14]. Thermoplastic materials have also been introduced for the fabrication of PRs using the indirect technique [15]. Computer-assisted design/computer-assisted machining (CAD/CAM) allows for the milling of 3D-designed objects from bulk material, and this technique has been reported to provide high precision [16]. PRs fabricated using CAD/CAM are said to have better color stability and more precise marginal quality than resin that has undergone conventional processing, which includes hand mixing, molding, adapting, gross finishing, and so on [17]. 3D printing is another technology that is used in dentistry to fabricate PRs, dental implants, orthodontic models, metal restorations, implant surgical guides, and other products [18]. These technology-sensitive materials require special equipment and are used for rapid prototyping, including liquid-based stereolithography and powder-based 3D printing [19].

The successful performance of a provisional material is not based solely on its mechanical properties, but also on its interaction with its immediate environment. Therefore, other factors such as marginal adaptation, color stability, and pulp and gum response need to be assessed [20]. Up to date, no type of PRM has been developed to eliminate the discoloration under various staining media. Therefore, this subject is still controversial. Several studies in various staining media were conducted. However, the color stability of PRMs in khat extract medium has never been investigated. Rayyan et al. [15] concluded that the CAD/CAM milling PRM demonstrated the best color stability and mechanical properties and may be used for long-term provisional restorations. Atria et al. [33] concluded that the PMMA CAD/CAM milling PRM was the best color stable. PMMA auto-polymerized acrylic resin and Bis-acrylic SC composite performed less well than PMMA CAD/CAM milling PRM. 3D printing PRM was the least stable in color and decreased significantly over time. Song et al. [34] concluded that the color stability of conventional materials varied depending on the staining solution. PMMA milling blocks showed a relatively low color change up to 4 weeks, but the color change significantly increased after 8 weeks. 3D-printed materials exhibited a high color change or a significant increase in color over time. P. Malik and M. Rathee [2] concluded that Bis-acryl composite resins (Integrity and Luxatemp Fluorescence) were more color-stable than the auto-polymerized acrylic resin (DPI self-cure tooth molding powder, Unifast Trad). Guler et al. [37] concluded that the methyl methacrylate-based PR material (TemDent) was found to be more color-stable than the Bis-acryl composite resin (Protemp II and Luxatemp) and light-polymerized (Revotek LC) composites that were tested. Sham et al. [10] concluded that the Bis-acryl composite resins (Luxatemp and Integrity) were the most color-stable PRMs tested, compared to the methyl and ethyl methacrylate-based resins. Yannikakis et al. [21] found that the composite-based materials, especially the light-curing composite materials, were the least color-stable in their study.

Researchers have concluded that practically all provisional restorative materials only exhibit satisfactory color stability for a short period of time and that, when subjected to staining solutions, they all eventually discolor [21]. There is no single material available right now that meets the optimal requirements for all situations. However, certain materials have already been productively employed for this purpose [9]. That is why new materials are being produced and introduced to the market in an attempt to improve these materials or reduce their defects.

Yemeni society, like any other, has its own unique traditions and customs. One of the most common behaviors among Yemenis that could affect the color of an aesthetic restoration is chewing khat. There has been little research on how chewing khat affects tooth discoloration. According to various studies, teeth stains can come from a variety of extrinsic sources, including khat in the Horn of Africa and tobacco and tea in the Arabian Peninsula [22]. Over 20 million people chew fresh leaves from khat trees (Catha edulis Celestrasae) every day in Yemen and other East African countries. Khat (qat) chewing is a popular social behavior that has spread to Yemeni, Somali, or East African communities in the United States and the United Kingdom [23]. In Yemen, there are many different varieties of khat, including Al-Arhabi, Al-Hamdani, Al-Dhalea, Al-Ansi, Al-Qutini, Al-Muraisy, Al-Sabri, Al-Hattabi, Khat Mawia, and others. These names are clearly derived from the location of the tree, unlike other varieties of khat, such as Al-Sawti, Al-Thuhla, Al-Ballut, Al-Shami, etc., whose names do not appear to be related to the location of the tree.

Since no previous studies have been published on the color stability of PRs in khat extract medium, this topic was chosen for this study. The goal of this study was to assess the impact of khat extract and the duration of immersion time on the color stability of the tested PRMs and to demonstrate the differences in color changes between them. Two null hypotheses were made in this study. The first hypothesis is that there are no significant differences in color change between the tested PRMs in the immersion media. The second hypothesis is that an increase in the immersion period does not have a significant impact on color change.

## Method

The study was an in vitro observational study. Five different PRMs were used to fabricate 50 disc-shaped specimens with a diameter of 10 ± 0.3 mm and a thickness of 2 ± 0.3 mm. The properties of the tested PRMs are shown in Table 1. The dimensions of the specimens were checked using an electronic caliper. A2 shade was confirmed using a VITA Easyshade V Spectrophotometer for all the tested PRMs. Ten specimens of each PRM were prepared.

**Table 1:** Properties of the tested PRMs

Product	Type	Fabrication Method	Manufacture	Lot no.
TAKILON BB	(PMMA) SC Acrylic Resin	P/L Hand Mixing	SPD Co. Italy	032A0508
TRANSCEN TEMP	LC Composite	Moldable Putty	Nexobio Co. Korea	TT200409
PRIME CROWN	Bis-Acrylic SC Composite	Auto-mix Syringe	Prime Dente Co. U.S.A	VNG22W
CERAMILL TEMP	(PMMA) Block	CAD/CAM Milling	Amann Girrbach Co. Austria	45,017
DENTAL SAND	Liquid Resin	3D Printing	Harzlabs Co. Russia	

Experimental research complied with relevant institutional, national, and international guidelines and legislation. Ethical approval form number 828 was obtained from the Medical Ethical Committee of the Medical Researches, Faculty of Dentistry, Sana’a University, Yemen.

### Manually fabricated specimens

Thirty specimens were fabricated of PMMA self-cured acrylic resin (Takilon bb), light-cured composite (Transcend Temp), and Bis-acrylic self-cured composite (Prime Crown) PRMs.

**Fig. 1:**
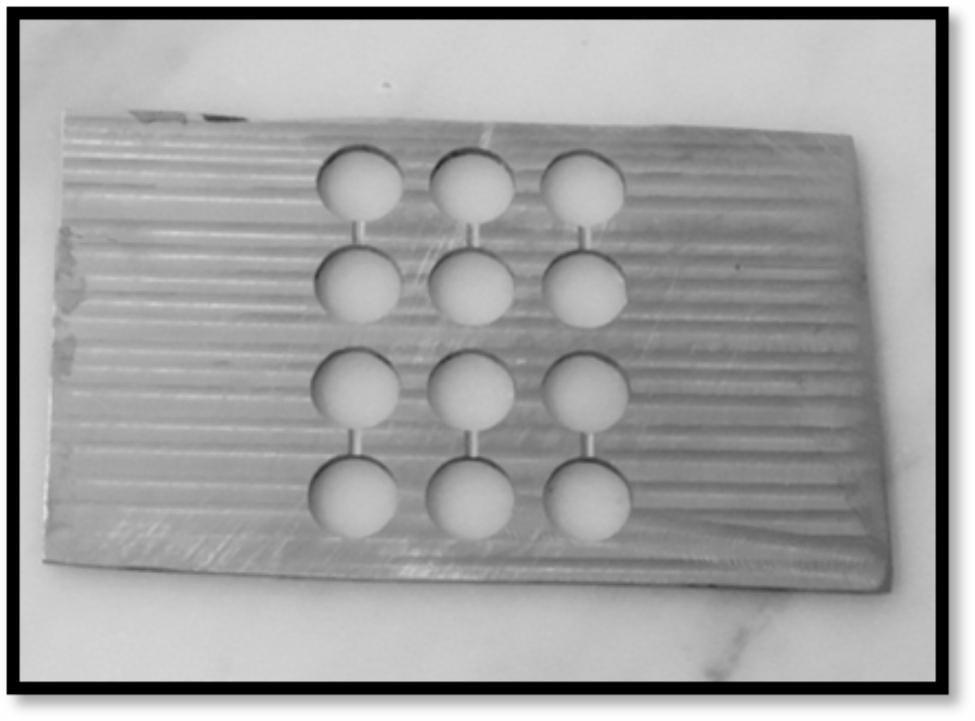
Metallic mold fabricated by a CNC machine showing the molding holes and the groove that connects each two holes

The specimens were prepared using a metallic mold that was fabricated by a CNC machine. To provide better distribution of the material and avoid porosity formation, during the process of pouring the material into the mold, each two holes were connected to each other by a shallow groove on each side (Fig. 1). The material was placed in the metallic mold, covered by a polyester stripe, and held between two glass slabs with a constant load for 30 s. After the material had completely set according to the manufacturer’s instructions, the specimens were removed from the mold.

For LC composite PRM (Transcen Temp) specimens, each surface was cured twice by an LED light curing unit (LY-B200, NO: 181,230,020). The first curing was done about 2 mm away from the polyester strip, followed by a second curing about 1 mm away from the surface of the specimen for 20 s each, as per the manufacturer’s instructions. Each specimen was prepared separately to avoid the light curing effect on the other specimen and then removed from the mold.

Specimens were checked. Any specimen with any abnormality, such as porosity, or that did not meet the dimensions was excluded and replaced with an accepted specimen.

### Digitally fabricated specimens

Disc-shaped specimens with a diameter of 10 mm and a thickness of 2 mm were designed using a 3D modeling software program (Ceramill Mind v2.4-7228) and exported as a file in Standard Tessellation Language (STL) format to two separate milling and 3D printing machines.

Ten specimens of the CAD/CAM milling PRM (Ceramill Temp) were fabricated using a milling machine (AMANN GIRBACH, Model: Ceramill Mikro (5X), SN: AAC39032, Lot: AWE179) with a constructing file (Ceramill TEMP 11- SlotAdapter 712,021.nest).

Ten specimens of the 3D printing PRM (Dental Sand) were fabricated using a DLP (Digital Light Processing) 3D printing machine (Phrozen Sonic XL 4 K Desktop 3D Printer, Model: Phrozen Sonic XL 4 K, Year: 2020) with a resolution of 50 μm and a constructing file (SliceJob_PhrozenSonicXL4K.zip). To remove the excess liquid resin, the specimens were dried with compressed air and washed in an ultrasonic bath with the solvent cleaner for 2 cycles of 3 min, as per the manufacturer’s instructions. After that, the specimens were post-cured in an oven with a turntable and 36–48 W UV LED lamps (Phrozen UV Light Post-Curing, Model: PC-60-DJ, Year: 2020) according to the manufacturer’s instructions.

### Surface treatment

To standardize the surface of all different specimens, Dian Fong polishing burs (from Dian Fong Abrasives Co. Ltd.) were used. A green sandstone bur with coarse pumice was used first, followed by a silicon bur, and then a rag wheel bur for 15 s each one, on each surface. A blind random method of polishing was applied during the polishing process.

### Khat extract preparation

Al-Arhabi khat, one of the most famous types of khat in Yemen, was purchased from the khat market in Sana’a city, Republic of Yemen. Initially, a suitable amount of soft fresh leaves and twigs of khat were cleaned and air-dried. Then, they were blended three times using a manual blender. After that, they were blended using an electric blender, pausing the blender every few seconds until the sound of the blender changed, which took about 15–20 s. Then, 20 g of the electrically blended leaves and twigs of khat were added to 500 milliliters of distilled water and shaken by an electrical shaker at 200 revolutions per minute for 5 h in a Napco incubator at 37 degrees Celsius. After that, the shaken material was filtered using string, medium-grade filter paper. Then, an aqueous khat extract was prepared by adding 100 milliliters of mineral water to 100 g of khat extract in a 1:1 ratio. The pH of this aqueous khat extract medium was 6.03 ± 0.23 (SD) at 37 degrees Celsius, which was within the normal limits of salivary pH (6–7), as stated by Humphrey and Williamson [24]. The researcher performed the same procedures daily to obtain a fresh solution.

### Pilot study

A pilot study was conducted with 20% of the specimens. Baseline measurements were taken after 24 h of immersion in distilled water at 37 °C. Then, the measurements were retaken after 24 h of immersion in aqueous khat extract and distilled water media at 37 °C. The variability of specimen preparation, the accuracy of the spectrophotometer, and the accuracy of specimen repositioning and re-measuring in the measuring mold were clear.

### Experimental procedures

First, the specimens were classified. Then, after 24 h of storage in distilled water at 37 °C, the baseline measurements were recorded. There were two subgroups of each PRM: one was immersed in the aqueous khat extract (treatment medium), and the other was immersed in distilled water (control medium). Distilled water was used in this study because artificial saliva was unavailable in the local market. The use of distilled water has been reported in previous studies, such as Sham et al. [10], Ergun et al. [7], and Rutkunas et al. [36].

Each of the five specimens in each subgroup was placed in a glass test tube with a label indicating the specimen number, the type of material, and the medium of immersion. The tube was concave, so that both surfaces of the specimen were facing the solution at all times, and were not touching the bottom of the tube. Then, all the tubes were held on a stand. After that, each specimen within the tube was submerged in the solution of aqueous khat extract (treatment medium) or distilled water (control medium), leaving 1 milliliter of the tube empty. Then, the tube was closed with a tightly sealed plastic cover to prevent the escape of gases. Next, the specimens within the solution in the tubes were stirred directly after immersion and then every 8 h for 10 min using a shaker (MX-T6-S) with 70 revolutions per minute (rpm). This was done to prevent air entrapment around the specimens and sedimentation of the solution. Finally, the immersion media along with the specimens were kept in the incubator at 37 °C at all times, and the temperature was checked with a thermometer.

To maintain the effect of fresh khat extract and to avoid bacterial or yeast contamination, the khat extract and distilled water solutions were renewed every 24 h for 7 days. The measurements were retaken after 1 day and 7 days of immersion using a spectrophotometer. Before each measurement, the specimens were rinsed under running distilled water, then dried with a clean, soft napkin, and air-dried in mild weather for 30 min. Each specimen was measured twice on each side, and the average value was calculated. The color change (ΔE*) was calculated using the color difference formula: ΔE* = [(ΔL*)^2^ + (Δ a*)^2^ + (Δb*)^2^]^½^. Khat extract and aqueous khat extract pH were measured twice by a pH meter every day during the week of the experiment, and the mean pH for each one was collected separately.

### Measuring mold

**Fig. 2:**
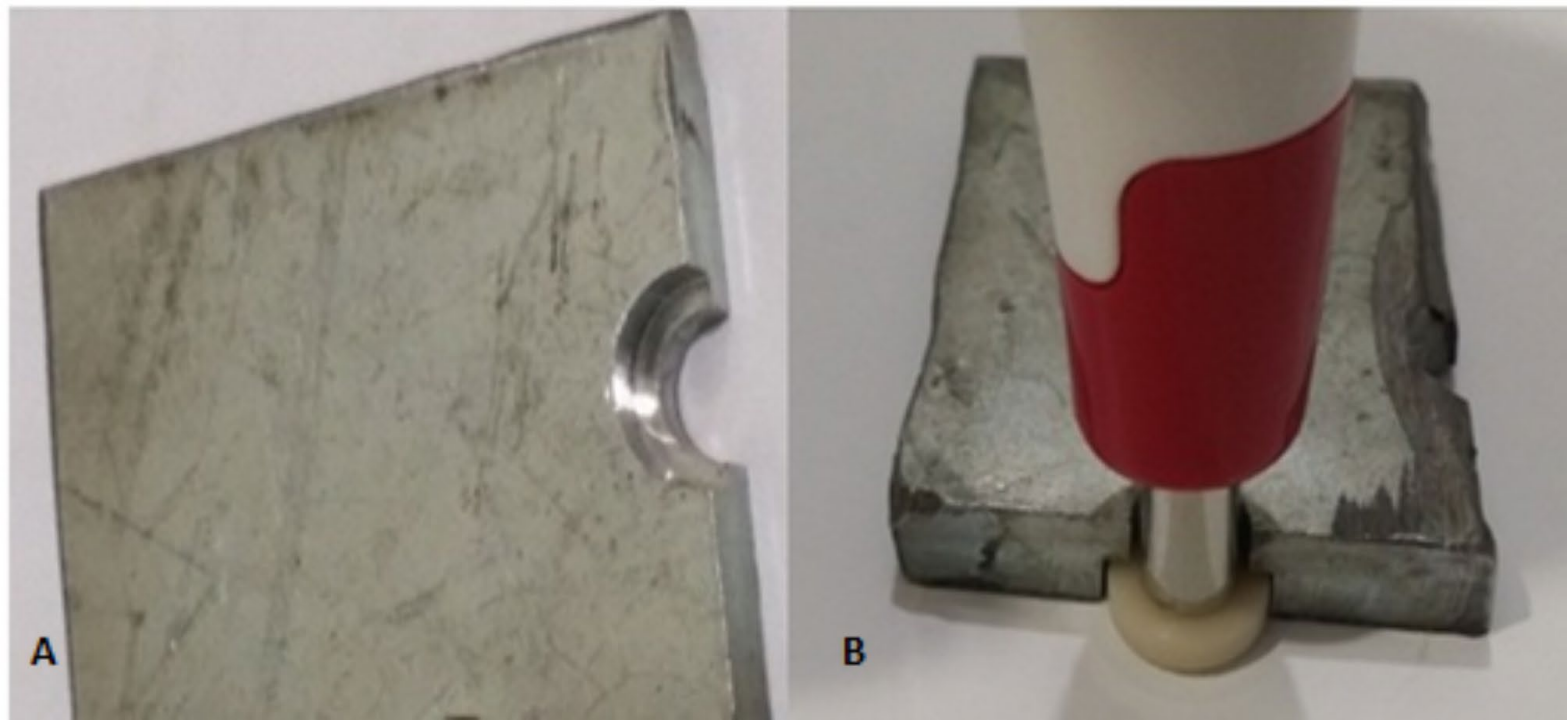
**(A)** Measuring mold fabricated by a CNC machine; **(B)** Specimen in the mold with a spectrophotometer centered over it for the process of color measurement

To standardize the point of re-measuring over the surface of the specimen, a metallic mold was fabricated by a CNC machine (Fig. 2). A half-moon-shaped chamber was prepared according to the radius of the specimen. This chamber can accommodate one half of the specimen, with one of the specimen surfaces facing the roof of the chamber and the other surface facing a white background. A semicircular opening was prepared in the midpoint of the roof of the chamber, according to the radius of the spectrophotometer tip. This ensures that the measuring point on the surface is approximately unified with each measurement, even if the specimen is rotated in the mold.

### Statistical analysis

The independence t-test was used to compare the quantitative data of each material between both media. A paired t-test between ΔE*^1^and ΔE*^2^ of each PRM was used to evaluate whether there is a significant change in color with an increase in the duration of immersion time. A three-way ANOVA was used to test the significance of the variables involved (PRMs, media, and immersion time) and their interactions. A one-way ANOVA was used to test the significance of color change among the tested PRMs at every measurement time. The Bonferroni test was used for post-hoc multiple comparisons. A p value (p < 0.05) was considered statistically significant.

## Results

The reliability test was performed after two weeks of reexamination of 10% of the specimens. Cronbach’s Alpha value of 0.997 indicates a high level of internal consistency for our sample’s data.

**Fig. 3:**
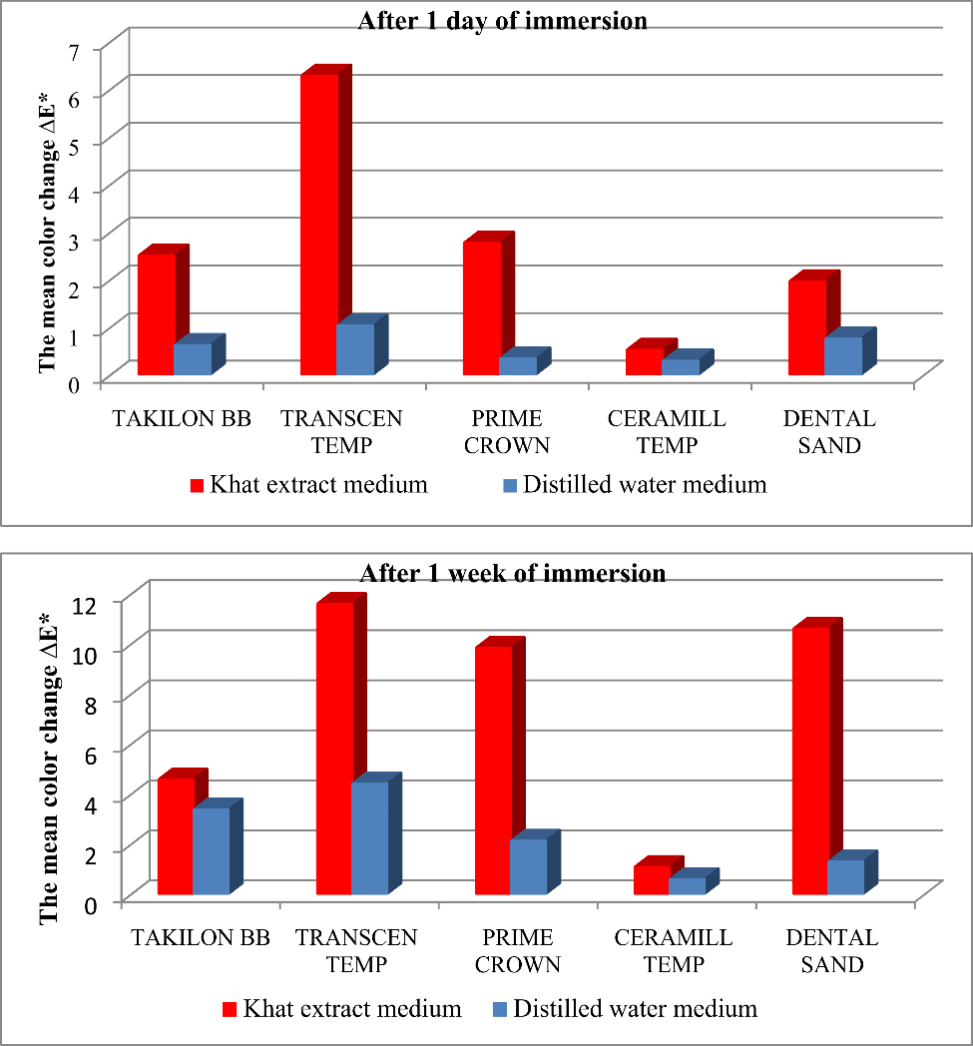
Graphical representation of the mean color change (ΔE*) of the tested PRMs after 1 day and 1 week of immersion

Khat extract was a statistically significant (p < 0.05) factor affecting the color stability of the tested PRMs, except for PMMA SC acrylic resin, where this effect was statistically insignificant after 1 week of immersion compared to that at 1 day. The duration of immersion time was also a statistically significant (p < 0.05) factor, increasing the color change of the tested PRMs. There was a statistically significant difference in color change among the tested PRMs (p < 0.05). The results are summarized in Table 2 and illustrated in Fig. 3.

**Table 2:** The mean color change (ΔE*) of tested PRMs in khat extract and distilled water media

Material	S.G	N	One Day			One Week		
			∆E* Mean ± SD	T Test	P Value	∆E* Mean ± SD	T Test	P Value
**TAKILON BB**	Khat	5	2.5 ± 0.56	6.1	0.001	4.58 ± 1.25	1.3	0.2
	water	5	0.62 ± 0.41			3.38 ± 1.68		
**TRANSCEN TEMP**	Khat	5	6.28 ± 0.51	17.9	0.001	11.65 ± 0.87	8.1	0.001
	water	5	1.03 ± 0.41			4.42 ± 1.79		
**PRIME CROWN**	Khat	5	2.77 ± 1.17	4.67	0.009	9.84 ± 1.35	12.51	0.001
	water	5	0.34 ± 0.06			2.13 ± 0.29		
**CERAMILL TEMP**	Khat	5	0.52 ± 0.17	2.47	0.04	1.08 ± 0.31	2.89	0.02
	water	5	0.29 ± 0.13			0.59 ± 0.22		
**DENTAL SAND**	Khat	5	1.96 ± 0.78	3.39	0.02	10.67 ± 1.77	11.5	0.001
	water	5	0.76 ± 0.15			1.30 ± 0.45		

S.G = Subgroup, N = Number of specimens, SD = Stander Deviation, ΔE* = color change

### Differences in color changes after 1 day of immersion in khat extract (ΔE*^K1^)

The LC composite demonstrated the highest color change, with a mean of ΔE*^K1^ 6.28, which was statistically significant (p < 0.05) when compared to each of the other tested PRMs. PMMA CAD/CAM milling PRM demonstrated the least color change, with a mean of ΔE*^K1^ 0.52, which was also statistically significant (p < 0.05) when compared to each of the other tested PRMs.

Bis-acrylic SC composite, PMMA SC acrylic resin, and 3D printing PRM demonstrated statistically insignificant color change between them, with mean values of ΔE*^K1^ 2.77, 2.5, and 1.96, respectively.

### Differences in color changes after 1 week of immersion in khat extract (ΔE*^K2^)

The LC composite demonstrated the highest color change, with a mean of ΔE*^K2^ 11.6, followed by 3D printing PRM and Bis-acrylic SC composite, respectively, with a mean of ΔE*^K2^ 10.6 and 9.84. The difference between these three PRMs was statistically insignificant. PMMA CAD/CAM milling PRM demonstrated the least color change, with a mean of ΔE*^K2^ 1.08, which was statistically significant (p < 0.05) when compared with each tested PRM.

Among the manually fabricated PRMs, the PMMA SC acrylic resin demonstrated the best color stability, with a mean of ΔE*^K2^ 4.58. This was statistically significant (p < 0.05) when compared to that of each manually fabricated PRMs.

### Differences in color changes after 1 day of immersion in distilled water (ΔE*^W1^)

The LC composite demonstrated the highest color change, with a mean of ΔE*^W1^ 1.03. 3D printing PRM and PMMA SC acrylic resins followed, with means of ΔE*^W1^ 0.76 and 0.62, respectively. The difference in color change between these two PRMs was statistically insignificant. PMMA CAD/CAM milling PRM demonstrated the best color stability among all tested PRMs, with a mean of ΔE*^W1^ 0.29 and the difference was statistically significant (p < 0.05) when compared to the LC composite but not when compared to the other PRMs.

Bis-acrylic SC composite, with a mean of ΔE*^W1^ 0.34, was more stable in color than LC composite and PMMA SC acrylic resin. The difference between Bis-acrylic SC composite and LC composite was statistically significant (p < 0.05), while it was statistically insignificant with PMMA SC acrylic resin.

### Differences in color changes after 1 week of immersion in distilled water (ΔE*^W2^)

The LC composite demonstrated the highest color change, with a mean of ΔE*^W2^ 4.42, and was statistically insignificant when compared to PMMA SC acrylic resin, with a mean of ΔE*^W2^ 3.38. However, it was statistically significant (p < 0.05) when compared to each of the other tested PRMs. PMMA CAD/CAM milling PRM demonstrated the best color stability among all tested PRMs, with a mean of ΔE*^W2^ 0.59. It was statistically significant (p < 0.05) when compared to LC composite and PMMA SC resin, but was not statistically significant when compared to Bis-acrylic SC composite and 3D printing PRM, with a mean of ΔE*^W2^ 2.13 and 1.3, respectively.

PMMA SC acrylic resin demonstrated a statistically significant higher color change (p < 0.05) than PMMA CAD/CAM milling PRM, but was not statistically significant when compared to the other two PRMs. Bis-acrylic SC composite demonstrated statistically insignificantly higher color change, with a mean of ΔE*^W2^ 2.13, when compared to 3D printing and PMMA CAD/CAM milling PRMs.

## Discussion

According to the results of the present study, the null hypotheses were rejected. There were significant differences in color change among the five tested provisional restorative materials (PRMs). The tested PRMs showed a considerable increase in color change as the immersion time increased. The results of this study demonstrated that khat extract has a high staining ability on the five PRMs tested. The difference in composition of the tested materials and the duration of immersion time were shown to be factors affecting the degree of color change of these tested PRMs.

The color stability of PRMs in tea, coffee, red wine, juices, and other substances has been studied in a number of studies. However, the color stability of provisional restorations (PRs) in khat extract media has not been previously studied. Khat chewing is a common social behavior in Yemeni daily life. Khat is widely cultivated in parts of East and South Africa, the Arabian Peninsula, Ethiopia, Kenya, Yemen, Somalia, Sudan, and Madagascar. It has also been found in Afghanistan and Turkistan [25].

In the present study, a color change expressed by a ΔE* ≥ 3.3 was considered visually perceptible and clinically unacceptable [3, 26–28]. A ΔE* < 3.3 was considered clinically acceptable, either imperceptible (ΔE*≤ 2) [21] or just perceptible (2 < ΔE* < 3.3) [29]. The findings of this study showed that the khat extract medium had strong staining power. This agreed with the findings of Al-Anesi et al. (2019) [30], with the exception that their materials were composite restorative materials. It also agreed with the findings of Yarom et al. [31], who found that 91.2% of khat chewers had stained teeth, while no stains were seen in the control group. This is because their research was conducted in vivo on real teeth. Tannins and a small amount of fluoride are present in crude khat, which may be the cause of the staining [32].

At 1 day and 1 week of immersion in khat extract media, PMMA CAD/CAM milling PRM was statistically significantly different from all other investigated PRMs in terms of color stability, with negligible color change. The LC composite PRM was the least stable in color among all the tested PRMs, with unacceptable color change. This color change was statistically significant when compared to each of the tested PRMs at 1 day, but after 1 week of immersion, the color change was insignificant when compared with 3D printing and Bis-acrylic SC composite PRMs. The color stability of LC composite was found to be the lowest, followed by that of 3D printing and Bis-acrylic SC composite PRMs, with the differences between them being negligible. Among the manually fabricated PRMs used in this study, PMMA SC acrylic resin had the most stable color, displaying barely perceptible color change after 1 day and being statistically less significant than Bis-acrylic SC composite PRM. However, after 1 week of immersion in khat extract medium, it showed an unacceptable color change that was statistically less significant than LC composite and Bis-acrylic SC composite PRMs.

The results of this study were consistent with those of Atria, Lagos et al. (2020) [33]. However, their study did not include LC composite PRMs, and the samples were subjected to 6,000 cycles of thermocycling (TC) in a water bath between 5 and 55 °C with a dwell time of 30 s in each bath before the color stability was determined. The results of this study were also in agreement with Song et al. [34]. However, they used coffee and black tea as staining media, and the measurements were taken at 1, 2, 4, 8, and 12 weeks. This was also in agreement with Shin et al. [35] on the CAD/CAM fabrication of PRMs, milling, and 3D printing. However, their measurements were obtained at 2, 7, and 30 days, and their media consisted of distilled water, grape juice, coffee, and curry.

Among the manually fabricated PRMs, LC composite, Bis-acrylic SC composite, and PMMA SC acrylic resin, the results of this study were in agreement with Rutkunas et al. [36]. However, their study used food colorants, coffee with sugar, and red wine as staining media. This study was also in agreement with Guler et al. [37]. However, they used tea as a staining medium, and ΔE* was evaluated after 48 h. Finally, this study was in agreement with Yannikakis et al. [21]. However, their media were coffee and tea, and they used dual-cured composite instead of LC composite.

The findings of this study corroborated those of Shin et al. [35]. They concluded that although water sorption is only one contributing cause to the low color stability of 3D printing PRMs, it cannot fully explain it. The DLP method uses a micro mirror 3D printing principle, which results in a slightly more distinctive pattern on the surface. This may have an impact on color stability. Another contributing factor to the low color stability is the low polymerization rate of 3D printing resins compared to other materials [35]. Another potential contributing factor to the low color stability of 3D printing PRMs is the presence of oxygen- inhibiting layers.

The color shift of the studied PRMs in distilled water was caused by water sorption. Water absorption can also affect the optical properties of PRMs [10]. The color and optical properties of provisional restorative materials have been shown to be affected by water accumulation and photo-oxidation [36, 38]. These may be the causes of the color changes of the specimens upon immersion in distilled water.

Intrinsic and extrinsic discolorations combine to produce color changes. Intrinsic discoloration can occur when pigments penetrate through microcracks or interfacial gaps at the filler-matrix contact. Extrinsic discoloration can occur when polar pigments and colorants present in the media are adsorbed onto the surface of resin composite materials [39]. The polarity of the colorants can influence the degree of penetration into the composite. More polar colorants tend to be adsorbed on the surface of the material, while less polar colorants may be more easily absorbed into the substance [26]. Both adsorption and absorption of colorants can be used to describe the staining process. The latter phenomenon, stain sorption, is closely connected to water sorption. According to a number of studies, filler content, the existence of leftover unpolymerized monomers, the presence of air bubbles, and the degree of cross-linking of resin molecules all have an impact on how much water is absorbed [36].

Depending on their chemistry and structure, dental polymers exhibit hygroscopic and hydrolytic effects to varying degrees. Fillers can have a significant impact on the uptake and dissolution of solvents in a polymer network, probably in proportion to their proportion, as fillers diminish the overall volume of the absorbing polymer. The main determinants of water sorption are the density of the dental polymer network, the hydrogen potential, and polar interactions. Dental monomers contain hydrolytically sensitive groups such as hydroxyl, ester, and urethane bonds. Although these monomers and the polymers they give rise to are not particularly hydrophilic, they do absorb water to an extent that is harmful [40].

PMMA blocks of the CAD/CAM milling PRMs are made by polymerizing in a high-temperature and high-pressure environment. Therefore, the polymerization rates in these materials are high, and their structures are compact [35]. The polymerization rate is the velocity and degree of the reaction. A higher polymerization rate leads to higher curing and higher mechanical properties. The chemical composition and unique feature of the complete processing of prefabricated PMMA blocks of the CAD/CAM milling PRMs may be the reason they are the most stable in color. The photoinitiator component, resin matrix composition, light-curing device, and irradiation time are all factors that affect the color stability of light-cured composite materials [36].

The tested PRMs, the medium, and the immersion time all had substantial interactions. This interaction was revealed by a change in the color of the tested PRMs. All of the tested PRMs showed a statistically significant increase in color change when the immersion period was extended from E*1 (immersion for 1 day) to E*2 (immersion for 1 week). This is because the PRMs absorbed and adsorbed more of the media over time, making the length of immersion time a significant factor that influences the intensity of this interaction.

It was observed that the color of the 3D printing material changed surprisingly more after one week of immersion in khat extract medium. This suggests that with further research, this material may become the least stable in color if the immersion period is extended beyond the duration of this study. Atria, Lagos et al. [33] reported that 3D printing PRM has the least color stability and degrades drastically over time. A week of immersion in the khat extract medium did not statistically change the color of PMMA SC acrylic resin PRM when compared to its color change in distilled water. This suggests that the effect of khat extract on the color stability of this PRM decreased as the substance began to become saturated with pigments.

Al-Alimi et al. [41] reported that the pH of khat extracts is 5.3. In this study, the mean pH of khat extract was 5.5 with a standard deviation of 0.29. The pH of the aqueous khat extract used in this study was 6.03 with a standard deviation of 0.23 (SD) at 37 °C. This pH of the aqueous khat extract medium was within the normal range of salivary pH, which is typically 6 to 7, as reported by Humphrey and Williamson [24]. The aqueous khat extract medium was created by adding 100 ml of mineral water to 100 g of khat extract in a 1:1 ratio. The pH of the mineral water used (a Shamlan bottle) was 7.1. The acidic components in khat are responsible for its low pH. Khat leaves contain 257.20 mg of ascorbic acid per 100 g [41]. Studies have shown that khat leaves contain tannins in the range of 3.5 g/100 g to 9.7 g/100 g [42]. The interaction of astringent substances, such as tannins, with salivary proteins and glucosamine glycan (mucopolysaccharides) is thought to be the primary mechanism causing the decrease in salivary lubricity [43]. Khat chewers are more likely to experience attrition, staining, tooth caries, swelling of the salivary gland, and inflammation of the parotid duct [44].

This study has some shortcomings. While clinical PRs will have an uneven shape with convex and concave surfaces, the specimen surfaces in this study were flat. In this study, khat extract medium was used to assess the products’ color stability. However, provisional materials may come into contact with other food-staining agents in the oral environment. The degree of overall color change may also be influenced by additional factors such as heat cycling and abrasion. Future studies should take these things into account.

In this study, five specimens (n = 5) were immersed in each medium. This number of specimens was consistent with the study by P. Malik and M. Rathee [2]. Color stability was evaluated by accelerated aging for five specimens of each tested PRM [28]. In other studies, the number of specimens immersed in each medium was higher. For example, Sham et al. [10], Ergun et al. [7], Guler et al. [37], and Shin et al. [35]. Therefore, the sample size in this study could be considered a limitation that should be taken into account in future studies. Finally, although 7 days of specimens’ immersion brought significant results, this duration seems a bit short. Therefore, further studies could be conducted for a longer period of time.

## Conclusion

Under the limitations of the current study, it can be concluded that khat extract has a high staining ability on the tested PRMs. PMMA CAD/CAM milling PRM was the most stable in color among all the tested PRMs. However, LC composite PRM was the least stable in color. The increase in immersion time was a significant factor in the color change of the tested PRMs. The color of the 3D printing PRM was the most affected over time.

## Data Availability

The datasets generated and/or analyzed during the present study are not publicly available, as ethics approval was granted on the basis that only the researchers involved in the study could access the identified data. However, the data are available from the corresponding author upon reasonable request.

## Abbreviations

PR: Provisional Restoration
PRMs: Provisional Restorative Materials
PMMA: Poly-Methyl Methacrylate
LC: Light Cured
SC: Self-Cured
CAD/CAM: Computer-Assisted Design/Computer-Assisted Machining
3D: 3 Dimensional
CNC: Computer Numerical Control

## References

[1] Patras M (2012). Management of provisional restorations’ deficiencies: a literature review. J Esthetic Restor Dentistry.

[2] Rathee M (2010). Evaluation of color stability of temporary fixed partial denture materials: In–Vitro study. The Internet Journals of Dental Science.

[3] Doray PG, Li D, Powers JM (2001). Color stability of provisional restorative materials after accelerated aging. J Prosthodont.

[4] Haselton DR, Diaz-Arnold AM, Dawson DV (2005). Color stability of provisional crown and fixed partial denture resins. J Prosthet Dent.

[5] Burns DR, Beck DA, Nelson SK (2003). A review of selected dental literature on contemporary provisional fixed prosthodontic treatment: report of the Committee on Research in fixed prosthodontics of the academy of fixed prosthodontics. J Prosthet Dent.

[6] Kohli S (2017). Discolouration of Polymethyl Methacrylate versus Bisâ Acrylic based Provisional Crown and Bridge Dental Resins: Effect of Storage Media and Duration. Annals of Medical and Health Sciences Research.

[7] Ergun G (2005). In vitro color stability of provisional crown and bridge restoration materials. Dent Mater J.

[8] Kadiyala KK (2016). Evaluation of flexural strength of thermocycled interim resin materials used in prosthetic rehabilitation-an in-vitro study. J Clin Diagn Research: JCDR.

[9] Kurtzman GM, Strassler HE, Fadm F (2006). Provisional fixed restorations. Dent Econ.

[10] Sham AS (2004). Color stability of provisional prosthodontic materials. J Prosthet Dent.

[11] Darshana P, Munde MU (2016). *Effect of indian chromatogens on color stability of commerically avaliable different tooth colored provisional restorative materials:an in vitro study* International. J oral care Res.

[12] Haselton DR, Diaz-Arnold AM, Dawson DV (2004). Effect of storage solution on surface roughness of provisional crown and fixed partial denture materials. J Prosthodontics: Implant Esthetic Reconstr Dentistry.

[13] Singh A, Garg S (2016). Comparative evaluation of Flexural Strength of Provisional Crown and Bridge Materials-An Invitro Study. J Clin Diagn Research: JCDR.

[14] Anusavice KJ (2012). S.C.a.R.H. In: Philips’ Science of Dental materials.

[15] Rayyan MM (2015). Comparison of interim restorations fabricated by CAD/CAM with those fabricated manually. J Prosthet Dent.

[16] Güth J, Silva JAe, Edelhoff D (2012). Enhancing the predictability of complex rehabilitation with a removable CAD/CAM-fabricated long-term provisional prosthesis: a clinical report. J Prosthet Dent.

[17] Stawarczyk B (2012). Load-bearing capacity of CAD/CAM milled polymeric three-unit fixed dental prostheses: effect of aging regimens. Clin Oral Invest.

[18] Dawood A (2015). 3D printing in dentistry. Br Dent J.

[19] Turbush SK, Turkyilmaz I (2012). Accuracy of three different types of stereolithographic surgical guide in implant placement: an in vitro study. J Prosthet Dent.

[20] Astudillo-Rubio D (2018). Mechanical properties of provisional dental materials: a systematic review and meta-analysis. PLoS ONE.

[21] Yannikakis SA (1998). Color stability of provisional resin restorative materials. J Prosthet Dent.

[22] Koleoso D, Shaba O, Isiekwe M. *Extrinsic tooth discolouration in 11–16 year-old Nigerian children* Odonto-stomatologie tropicale = Tropical dental journal, 2004. 27(106): p. 29–34. 15536719

[23] Al-Motarreb A (2002). Khat chewing and acute myocardial infarction. Heart.

[24] Humphrey SP, Williamson RT (2001). A review of saliva: normal composition, flow, and function. J Prosthet Dent.

[25] Gelaw Y, Haile-Amlak A (2004). Khat chewing and its socio-demographic correlates among the staff of Jimma University. Ethiop J Health Dev.

[26] Um CM, Ruyter I. *Staining of resin-based veneering materials with coffee and tea*. Quintessence Int, 1991. 22(5). 1924691

[27] Ruyter I, Nilner K, Möller B (1987). Color stability of dental composite resin materials for crown and bridge veneers. Dent Mater.

[28] Doray PG (1997). Accelerated aging affects color stability of provisional restorative materials. J Prosthodont.

[29] Gross M, Moser J (1977). A colorimetric study of coffee and tea staining of four composite resins. J Rehabil.

[30] Al-Anesi WA et al. *Effects of khat extract and other staining media on color change of composite resins subjected to various polishing methods*. Mater Sci, 2019.

[31] Yarom N (2010). Oral manifestations of habitual khat chewing: a case-control study. Oral Surg Oral Med Oral Pathol Oral Radiol Endodontology.

[32] Gashawa A, Getachew T (2014). The chemistry of khat and adverse effect of khat chewing. Am Sci Res J Eng Technol Sci (ASRJETS).

[33] Atria PJ, Lagos I, Sampaio CS (2020). In vitro evaluation of surface roughness, color stability, and color masking of provisional restoration materials for veneers and crowns. Int J Comput Dent.

[34] Song S-Y (2020). Color stability of provisional restorative materials with different fabrication methods. J Adv Prosthodont.

[35] Shin J-W (2020). Evaluation of the Color Stability of 3D-Printed Crown and Bridge materials against various sources of discoloration: an in Vitro Study. Materials.

[36] Rutkunas V, Sabaliauskas V, Mizutani H (2010). Effects of different food colorants and polishing techniques on color stability of provisional prosthetic materials. Dent Mater J.

[37] Guler AU, Kurt S, Kulunk T (2005). Effects of various finishing procedures on the staining of provisional restorative materials. J Prosthet Dent.

[38] Seghi R, Gritz M, Kim J (1990). Colorimetric changes in composites resulting from visible-light-initiated polymerization. Dent Mater.

[39] Yazici AR (2007). The effect of curing units and staining solutions on the color stability of resin composites. Oper Dent.

[40] Ferracane JL (2006). Hygroscopic and hydrolytic effects in dental polymer networks. Dent Mater.

[41] Al-Alimi K et al. *Tannins acid, ascorbic acid and fluoride from khat chewing plant*. Int J Dent Oral Health, 2017. 3(4).

[42] Dhaifalah I, Santavy J (2004). Khat habit and its health effect. A natural amphetamine. Biomed Pap Med Fac Univ Palacky Olomouc Czech Repub.

[43] Al-Alimi K, Kasim NA, Ahmad R. *Demineralization potential of qat extracts at composite restoration interface*. J Dent Res, 2008.

[44] Alsharabi AKK. ORAL AND PARA-ORAL LESIONS CAUSED BY TAKHZEEN AL-QAT.(QAT CHEWING). University of Khartoum; 2002.

